# Experiences of rehabilitation coordination among people on sick leave with mental health problems

**DOI:** 10.1080/02813432.2024.2361242

**Published:** 2024-06-04

**Authors:** Kristin Lork, Louise Danielsson, Maria E. H. Larsson, Kristina Holmgren

**Affiliations:** Department of Health and Rehabilitation, Institute of Neuroscience and Physiology, Sahlgrenska Academy, University of Gothenburg, Gothenburg, Sweden

**Keywords:** Qualitative research, return to work, sick leave

## Abstract

**Purpose:**

Return to work often requires collaboration between different stakeholders. Rehabilitation coordination is a resource in coordinating efforts during sick leave to facilitate return to work. The purpose of the present study was to describe how people at risk for sick leave or on sick leave with mental health problems experienced rehabilitation coordination.

**Materials and Method:**

The study had a qualitative approach using qualitative content analysis as described by Graneheim and Lundman. Eleven semi-structured interviews were conducted with persons at risk for sick leave or on sick leave due to mental health problems and with experience of rehabilitation coordination.

**Results:**

The participants experience of rehabilitation coordination were described by the overarching theme *Building a bridge with many bricks between the person and society*. The theme was formed by four categories and eleven subcategories reflecting the complex context of rehabilitation coordination. The categories were *Collaboration in a new setting, Unburdened within certain limits, The way back to work is a joint project* and *Recognising challenges beyond the person.*

**Conclusions:**

People with mental health problems experienced rehabilitation coordination as a meaningful link between healthcare and work. However, rehabilitation coordination needs to be more recognised within healthcare to increase accessibility. It seems important that interventions are directed not only towards the person, but also include the workplace for a sustainable return to work.

## KEY POINTS

It is important to make rehabilitation coordination visible within primary health care and actively inform people on sick leave with mental health problems that it is an option, as they often have difficulties finding information. It will increase their accessibility and enable autonomous decisions.

A respectful interplay based on a person-centred care approach seems fundamental for rehabilitation coordination. The interplay with rehabilitation coordinators and with other stakeholders affects the sick leave process and all parties need to collaborate for a sustainable return to work.

Three-party meetings with the person on sick leave, the rehabilitation coordinator and the employer, as well as teamwork, may provide better conditions for return to work as this can ensure that all stakeholders are working towards prioritised goals.

Targeted interventions at the workplace seem to be important and rehabilitation coordination could be a valuable bridge between healthcare and work for creating sustainable conditions for return to work.

## Introduction

Mental health problems such as depression, anxiety disorders and exhaustion disorder cause suffering for the individual and represent a challenge for society. The World Health Organization (WHO) has estimated the global prevalence of all mental disorders to be 970 million, equivalent to 13% of the world’s population at any one time [1]. The most common reason for sick leave in Sweden today is mental health problems, accounting for almost half of all ongoing sick leave. With mental health problems sick leave is longer, and the risk of relapse is higher than in other illnesses [2]. Living with mental health problems challenges the return to work process further as decreased motivation may be a hinderance, and motivation is considered a vital part of successful return to work [3]. Moreover, the stigma surrounding mental ill-health may cause barriers towards returning to the workplace [4]. Living with mental health problems during sick leave and the subsequent return to work process requires effective coordination. The present study will explore the experiences of people with mental health problems regarding the coordination between sick leave and the transition back to work.

Return to work is a complex process with interplays between the person on sick leave and several stakeholders with different concerns and interests [5]. Health care providers, social insurance agencies and workplaces are involved, and collaboration is a precondition for successful return to work [6]. Rehabilitation coordinators, also referred to as return to work coordinators or case managers, have gained increased recognition as a valuable resource in supporting return to work after sick leave. Research concerning rehabilitation coordination is sparse but growing. Rehabilitation coordinators engage with various stakeholders and processes to facilitate return to work [7]. Their role is not clearly defined and may vary as rehabilitation coordinators can be found in different positions, such as in the human resources departments of large companies, in insurance agencies or in healthcare systems. Contextual differences also need to be considered regarding legislation, insurance systems and healthcare systems, making international comparisons problematic [8]. A recent review listed eleven return to work stakeholders for workers on sick leave due to mental health problems [9]. Return to work coordinators were recommended to ensure communication and coordination between different stakeholders including employers, insurance agencies and health care providers [9].

To facilitate collaboration in Sweden between persons on sick leave, employers, health care providers and other stakeholders, a law for the provision of rehabilitation coordination was enacted in 2020. Rehabilitation coordination in Sweden is provided by the healthcare system and it is today offered at all primary health care (PHC) centres [10]. According to the law, rehabilitation coordination should include personal support and internal and external collaboration. The law states that every region should offer rehabilitation coordination efforts to persons on sick leave but not which professionals that should provide this service [10]. In Sweden the coordination function is carried out by rehabilitation coordinators ensuring that proper efforts are made to improve health and facilitate return to work. As the skill requirements for working as a coordinator are not regulated, several professional backgrounds exists such as occupational therapist, physiotherapist, social worker, or nurse [11]. The patient’s consent and participation are emphasized [10] which is consistent with healthcare legislation underlining the respect for patients’ self-determination and integrity [12,13]. Respecting the person and their needs and preferences is a fundamental ethical principle in person-centred care that is reflected in these laws [10,12,13]. The need for rehabilitation coordination is usually assessed by the coordinator in collaboration with the general practitioner (GP) issuing sickness certificates [11]. In a national survey coordinators report that the personal support by information, counselling and establishing rehabilitation plans constitute a large part of the assignment [14].

While current evidence regarding return to work interventions is inconclusive [15–20], positive effects of rehabilitation coordination in terms of increased return to work and improved internal and external collaboration have been reported in Swedish PHC settings [21–23]. However, a recent review exploring return to work coordinators for persons with mental health problems showed that, in the few studies conducted to date, no beneficial effects on return to work could be concluded [24].

Workers’ own experiences of sick leave coordination in qualitative research have illuminated contextual constraints that may lead to a lack of coordination between different systems [25,26]. Qualitative studies concerning rehabilitation coordination in Swedish PHC for people with mental health problems [27,28] and multimorbidity [29] have been completed, two of which involved experiences from people on sick leave [28,29]. Successful implementation of rehabilitation coordination for people with mental health problems was based on a detailed intervention packaging, formalised coordinator qualifications and recognition of organisational factors [28]. People with multimorbidity experienced rehabilitation coordination interventions as important in all four dimensions included in the analyses: the healthcare system; the labour market and workplace system; the sickness insurance system; and the personal system. Lack of advice and coordination were associated with prolonged sickness absence and deteriorated health [29].

Qualitative research, as well as identifying meaningful outcomes other than time to return to work, are essential for future studies [24]. From the perspective of persons on sick leave with mental health problems, rehabilitation coordination needs to be further explored with qualitative methods within PHC settings. Understanding what people on sick leave with mental health problems experience as meaningful efforts is important for improvement of the sick leave process. In the present study we aim to explore and describe how people at risk for sick leave or on sick leave with mental health problems experience rehabilitation coordination in PHC.

## Materials and method

### Design

To capture experiences of rehabilitation coordination in the complex return to work process, this study employs a qualitative, inductive approach with semi-structured interviews, using qualitative content analysis (QCA) as described by Graneheim and Lundman [30,31]. This method is characterised by a hermeneutic approach based on the ontological assumption that phenomena have multiple ways of being interpreted and that understanding relates to context and the researchers´ subjectivity [31,32]. QCA focuses on differences and similarities in the text and on analysing both manifest and latent content. Furthermore, the method permits flexibility regarding abstraction and interpretation levels, which was suitable as the participants´ narratives were expected to differ in depth and variation [30]. The inductive approach was considered appropriate as there are no background theories guiding the analysis, and as existing research in this area remains limited.

### Ethical considerations

The study complied with the ethical principles of the World Medical Association’s Declaration of Helsinki [33]. Informed written consent was obtained at the time of the interviews, and both oral and written information highlighted that participation was voluntary and could be terminated without any further consequences. The data was managed carefully and stored in a locked fireproof cabinet at the University of Gothenburg. Analyses were conducted with anonymised data as each participant was assigned a number, and results presented so that individuals could not be recognised.

### Recruitment and participants

Urban and rural PHC centres located in Region Vastra Gotaland, Sweden were contacted *via* email and telephone and asked if they wanted to inform eligible participants about the study. Ten out of 17 PHC centres agreed to help with the recruitment. Participants were patients with mental health problems such as depression, anxiety disorders and exhaustion disorder, who had experience of rehabilitation coordination. They were at risk for sick leave or were sick-listed full- or part-time. They should have taken part in at least three digital or physical meetings with their coordinator or with their coordinator and several stakeholders in collaboration. Exclusion criteria were high risk of suicide or addiction problems according to the treating health care professional.

Eligible participants received oral and written information about the study’s purpose from coordinators. If interested, participants were contacted by the researcher (KL) by telephone or mail. Fourteen participants were contacted, of which three participants declined to participate due to time constraints or mental health conditions. A total of eleven participants were interviewed. Most people of working age in Sweden are included in the sickness insurance system and a person can be on sick leave for 25%, 50%, 75% or 100% of the working time. The participants were on full or part-time sick leave except two participants of which one had recently returned to work and one received rehabilitation coordination as a preventative measure. A large majority had self-reported symptoms of exhaustion disorder [34] which is a clinical diagnose in Sweden similar to burnout in an international context, and two participants reported symptoms of depression (Table 1).

**Table 1.** Characteristics of the participants, *n* = 11.

Age	
25–40 years	3
41–55 years	6
56–66 years	2
Sex	
Women	10
Men	1
Marital status	
Single	4
Married/cohabitant	7
Self-reported symptoms	
Exhaustion disorder^1^	9
Depression	2
Sick leave	
0%	2
50%	3
75%	2
100%	4
Sick leave period	
<4 month	3
4-18 month	2
4–5 years	3
9–11 years	2
Educational level	
Higher education	6
Upper secondary education	5
Occupational class^2^	
High non-manual	4
Intermediate/low non-manual	6
Skilled/unskilled workers and self-employed	1

Note.

^1^ According to the Swedish version of International Classification of Diseases [ICD-10] [34].

^2^ The occupational classification is based on the International Standard Classification of Occupations [ISCO-08].

Participants represented a varied amount of contact with coordinators. Some had three to four meetings while others had three years of contact.

### Interviews

Participants could choose between a digital meeting *via* Zoom (*n* = 1) or a physical meeting at a research and development centre (*n* = 10). The interviews lasted between 36 and 72 min and were conducted between October 2021 and March 2022. One participant sent additional written notes which were included in the analysis. The interviews were audio-recorded and transcribed verbatim.

A semi-structured interview guide was developed [35], with questions relating to experiences of rehabilitation coordination and actions that had been taken or planned during the return to work process (Table 2). During the interviews, it was important to leave room for the participants’ experiences and reflections. They were encouraged to speak openly, and follow-up questions were used to deepen the understanding.

**Table 2.** Interview guide with examples of probes and follow-up questions.

Main probes	Suggested follow-up questions
What are your experiences of rehab coordination?	
How do you experience your contact with the rehab coordinator?	
How were you treated?	How did you get in touch with the rehab coordinator? What has your contact with the rehab coordinator looked like? How did you have contact? How often have you had contact?
Are there things that worked well?	If so, in what way?
Are there things that worked poorly?	If so, in what way?
Are there any interventions you missed?	If so, what are they?
What actions do you think are important to be able to work again?	
How do you want to be treated when you are on sick leave?	

### Coding and analysis process

The analysis was carried out according to Graneheim and Lundman’s description of QCA [30, 31] and the transcribed interviews formed the basis for the analysis. To get an overview and a sense of the material the analysis started with multiple readings of the interviews. In the next step, meaning units from three interviews were extracted and coded by two authors (KL, LD). Two additional interviews were analysed in the same way by three authors (KL, KH, ML). Coding was then compared and discussed among the authors. The analysis process continued with all data where meaning units were extracted and coded (KL). The codes were grouped and regrouped several times into subcategories and categories and finally a theme was formed. Continuous discussions among all authors regarding the analysis were held and the preliminary analysis was also discussed in a research group within occupational therapy and physiotherapy. Table 3 presents examples of the analysis from meaning units to category.

**Table 3.** Examples of meaning units, condensed meaning units, codes, subcategory and category.

Meaning unit	Condensed meaning unit	Code	Subcategory	Category
It has opened up a new, what do you say, view that there are things I can do if I want to and if I manage… my rehab coordinator, she knew exactly where we were and what I would need… She has been a great support to me all the way.	Rehab coordination opened up new views and provided support	Rehab coordination gave new views and support	Guidance provides empowerment and new perspectives	Collaboration in a new setting
Because I can´t do everything myself. For example, if it’s about the depression I've had, I may not see what she [the rehab coordinator] can see. She has more knowledge.	Cannot do everything myself in depression, do not see what rehab coordinator sees, who has knowledge	Rehab coordinator has other perspectives and knowledge		
To see it from another angle. So you can move forward, so you don´t go deeper into it… especially in my depression. But now she [the rehab coordinator] made me see something. I may not see everything yet, but a lot, that this I can do.	Can move forward from the depression and see things from another angle with the rehab coordinator	Rehab coordinator gave new angles		

## Results

### Building a bridge with many bricks between the person and society

The overarching theme *Building a bridge with many bricks between the person and society* reflected the complex context of rehabilitation coordination. The bricks represented the various stakeholders who were involved in the process to create a bridge between the person and society. The theme was based on the following four categories: *Collaboration in a new setting, Unburdened within certain limits, The way back to work is a joint project* and *Recognising challenges beyond the person.* These categories were developed through a total of eleven subcategories (Table 4).

**Table 4.** Overview of theme, categories, and subcategories of experiences of rehabilitation coordination.

**Theme** Building a bridge with many bricks between the person and society	
**Categories**	**Subcategories**
Collaboration in a new setting	Unknown and unclear task
	A cooperative link
	Guidance provides empowerment and new perspectives
	Straightforward but tactful interplay with risk of conflicts
Unburdened within certain limits	Shared responsibility
	Adaptation to the professionals
The way back to work is a joint project	Timely interventions with rehabilitation plans
	Better priorities with teamwork
	Identifying workplace support and barriers
Recognising challenges beyond the person	Stress leads to illness
	Sick leave may lead to feelings of guilt

### Collaboration in a new setting

The participants viewed rehabilitation coordination as something new and as the hub of collaboration between themselves and other people involved in their return to work, such as their GP, their manager, and the Swedish social insurance agency (SSIA). The encounter with a coordinator marked their initial experience of rehabilitation coordination, regardless of the duration of sick leave. The dynamics of rehabilitation coordination appeared unclear in relation to other stakeholders, with roles often muddled. Over time the coordinator was experienced as a guide who could show them new perspectives. The participants highlighted the need for clear roles, effective communication, and a supportive interplay between the coordinator and other stakeholders for a successful rehabilitation.

#### Unknown and unclear task

The function of rehabilitation coordination was perceived as unknown to the participants in the beginning of their sick leave process. They described not knowing that there were coordinators at the PHC center, or what they could help with. The participants wanted accessible information, to help them make decisions.

Because the rest of us do not know what opportunities there are and what people are working there at the health care centre. I know, there is a physiotherapist, but rehab coordinator or coordinator, I do not think many people know about that possibility. (Intermediate nonmanual worker, 58 years old woman with depression.)

The participants had been referred to rehabilitation coordination by their GPs. However, the participants suggested that all health care professionals should be able to propose rehabilitation coordination. Once the participants had established contact with the coordinator, the process was described as easy and adaptable to their individual needs.

The participants were uncertain about the role and boundaries of rehabilitation coordination, since there were many stakeholders involved. The lack of well-defined roles left the participants struggling with ambiguity, unsure of where to turn for assistance.

#### A cooperative link

The participants felt that the coordinator played a central role, like a spider in the web of all their contacts during the sick leave process. The coordinator served as a link between diverse stakeholders, both within the PHC centers and with external contacts. This link meant that information could pass more easily so that the participants did not need to recount their medical history or work situations repeatedly during different visits.

It is a transparency when the coordinator knows what is said from the SSIA, from the Public Employment Service, so the coordinator may forward it to my psychologist at the PHC centre and to my GP, so it has felt great and very safe to know this information goes straight in… And I do not have to rattle it off every time I have a visit to the GP or on regular visits to the psychologist. So that’s a clear advantage. (High nonmanual worker, 49 years old woman with exhaustion disorder.)

Since it was common among the participants to face challenges with memory and concentration due to their mental health, they appreciated that the coordinator adapted important information. This ability not only improved communication with GPs, particularly addressing misunderstandings with sick leave certificates, but also proved helpful in navigating external interactions with the SSIA.

The participants underlined the importance of continuity in their interplay with various stakeholders, with a particular emphasis on their relationship with GPs. Here, the coordinators were perceived helpful by providing support and information over time, especially since GPs sometimes changed.

#### Guidance provides empowerment and new perspectives

The participants emphasized that the function of rehabilitation coordination helped them to gain new insights and to increase their confidence, particularly when grappling with the complexities of healthcare and insurance systems.

It feels much easier (with rehabilitation coordination). It felt like you had someone on your side who helped you with things you can´t always process yourself when you are so tired and feel that your head doesn´t have the strength to concentrate properly. Then the coordinator is there and can help me by explaining how to do (things). (Unskilled worker, 39 years old woman with exhaustion disorder.)

The participants felt that it was demanding to navigate the healthcare and insurance systems when being on sick leave, and they appreciated the contact with the coordinator throughout the whole process. The coordinators, viewed as experienced guides, played an important role in steering them and providing support through the sick leave process.

According to the participants, it was important that the coordinator understood their whole work and life situation. The coordinator performed a comprehensive assessment, which they reflected on together. The participants described this assessment and reflection as they “assembled a puzzle”. As the pieces fell into places, the participants gained more clarity in an often chaotic sick leave period. The coordinators introduced and suggested interventions previously unknown to the participants and thus broaden their views. The coordinator’s ability to search for relevant information instilled confidence and the participants gained a sense of control over their situation.

Very good to have someone who has a little, yes, but control, and can say how things will develop, and I feel Ím confident that the rehab coordinator has the knowledge. It has worked well, and I feel Íve learned a lot about how things should work. It has made me feel safer and a little less nervous about returning to work. (High nonmanual worker, 28 years old woman with exhaustion disorder.)

#### Straightforward but tactful interplay with risk of conflicts

The quality of the interplay between participants and coordinators were important, according to the participants. They stressed the value of being respected and recognized as a whole person beyond their diagnosis.

The participants felt that the coordinator was committed to supporting them: one participant called their coordinator “a resolute warrior” while navigating interactions with the other stakeholders. By being assertive and focusing on facilitating, the coordinator pushed them forward in the rehabilitation process, said the participants. The delicate nature of this role required a constant balancing act, putting demands on the coordinators to convey calmness, listen attentively, and maintain a respectful attitude.

It must not be too gentle. We are not children, even if we become children when we feel ill, then we turn inwards. You (the rehab coordinator) listen and try to understand what that person needs… yes, listen is probably number one, maybe find a tempo that person has. (Intermediate nonmanual worker, 52 years old man with exhaustion disorder.)

Sometimes the balancing act did not work, resulting in a strained interplay between the participants and the coordinator, which in turn affected cooperation with other stakeholders negatively. Some participants felt patronized by the coordinator and at times treated like children in need of a reprimand.

It was very negative in the beginning. I don´t know how new the rehab coordinator was at work, but I felt that I had been on sick leave for so long, I know what it’s about. I felt like I was treated like a five-year-old. (Intermediate nonmanual worker, 47 years old woman with exhaustion disorder.)

Participants sometimes experienced being questioned by coordinators and not being listened to. If the relationship improved, the participants felt that their rehabilitation plans became more sustainable and realistic. Altogether, the participants described a supportive cooperation where everyone played to their strengths. Nevertheless, some encountered a lack of support from external stakeholders such as employers, unions, or the SSIA. Interactions with the SSIA were compared to police interrogations, with regulations often misunderstood, and a lack of communication causing time-consuming conflicts affecting the sick leave process.

The participants perceived a good interplay with coordinators as a basic precondition for cooperation with other stakeholders. However, if cooperation with other stakeholders did not work, rehabilitation coordination efforts were hindered.

### Unburdened within certain limits

The participants expressed a profound sense of personal responsibility in navigating the complexities of the sick leave process. While some found it challenging, considering it an “involuntary job”, they acknowledged that coordinators played a role in alleviating their burden by sharing certain responsibilities. However, this unburdening was not always easy, as the participants also felt responsible to meet the expectations of healthcare professionals.

#### Shared responsibility

Participants expressed a wish to learn better ways to cope and to manage returning to work. Participants who received rehabilitation coordination early in the sick leave process, described a notable shift when they were allowed to hand over some of the responsibility to the coordinator, for example seeking adequate rehabilitation interventions. This support encouraged participants to prioritize their own well-being in the sick leave process.

For me, it is to try to put myself a little more in the first place instead of everyone else. Because there’s a lot where I feel that no, I can´t let them pull away. I need to have time to rest. That’s what she (the rehab coordinator) has made me realise, I must think about myself too. (Unskilled worker, 39 years old woman with exhaustion disorder.)

#### Adaptation to the professionals

The participants recognized that healthcare professionals, including coordinators, might be under stress. The participants, being sensitive to stress, found themselves easily affected by others’ stress levels. Thus, they emphasized that it was important that the professionals were not showing signs of their own stress during the meetings.

When I was at the doctor’s, I think it was in spring. Then he had the work situation I had at work. I was just so (panting). He sat there and the phone rang and so it was the computer, it knocked on the door. We were going to talk and then there was a knock on the door again. I just, that was exactly what I had when I was working… So, I recognised it, so it just overwhelmed me right away. (Intermediate nonmanual worker, 57 years old woman with exhaustion disorder.)

In situations where coordinators or other professionals appeared stressed, participants held back to avoid adding to the professionals’ burden. This experience reveals a reversal of roles, with the participants feeling responsible for those intended to support them. The participants’ heightened sensitivity to stress influenced their own approach and the balance in the relationship between participants and professionals.

### The way back to work is a joint project

A key factor for sustainable return to work was described by the participants as joint planning involving all stakeholders, with the primary focus being on the workplace. Participants perceived rehabilitation coordination as the crucial link bridging between healthcare and work. The participants had different experiences of how rehabilitation coordination facilitated return to work, and they suggested interventions based on previous experiences.

#### Timely interventions with rehabilitation plans

The importance of timely interventions embedded within rehabilitation plans was emphasized to prevent a decline in motivation and self-esteem. If rehabilitation coordination was initiated late, the participants experienced that valuable time had been lost. They wanted equal access to rehabilitation opportunities, urging GPs and coordinators to actively suggest suitable interventions and provide rehabilitation “packages” tailored to the participant’s specific reasons for sick leave.

If you live in a big city, there are certain resources and then maybe you live in a very small town and then there is nothing… there should be a little, as I said, a small ready-made package with this type of support, so maybe you ask the patients "Now I notice that you have these symptoms. Maybe it would be good for you to take part in this or to take part in this.” (High nonmanual worker, 28 years old woman with exhaustion disorder.)

The participants emphasized that relevant interventions should be directed toward all daily activities, not only work. To maintain their motivation, the participants wanted to focus on realistic rehabilitation plans that were built upon their resources.

Coordinators played a key role in initiating rehabilitation meetings involving participants and other stakeholders. While the participants found these meetings valuable, the meetings also posed challenges, and a feeling of exposure. However, they perceived that support from the coordinators encouraged more open discussions about potential adaptations at work.

#### Better priorities with teamwork

The participants underlined that it was substantially different to have an entire team that included a coordinator, compared to only the individual contact with a GP. The experience of teamwork helped the participants to perceive a genuine rehabilitation process where all involved parties were supportive and actively engaged.

A long journey that is moving forward, but still very much support from the rehab coordinator, occupational therapist, psychologist and doctor and the work, it is the boss I have today. So that´s a team that helps me because I need to slow down for it to be sustainable. (Intermediate nonmanual worker, 48 years old woman with exhaustion disorder.)

The teamwork not only facilitated the return to work process but also saved time and energy. This efficiency was achieved through improved exchange of information and the establishment of common goals, enabling the participants to concentrate more on regaining their health. The participants described that the teamwork had a powerful influence and should be prioritized over individual interactions in rehabilitation and return to work.

#### Identifying workplace support and barriers

The participants emphasized that early workplace inclusion was important for a successful return to work. They regarded the coordinators as counselors, involved in facilitating adjustments and providing support at work, or suggesting changing work if necessary. While rehabilitation coordination was perceived as a link between healthcare and work, the participants recognized that their return to work depended on the workplace’s approach.

It was essential to investigate the workload and make suitable work adjustments before return to work. The participants emphasised that without workplace adjustments, rehabilitation interventions were pointless, and they risked returning to sick leave again.

The participants’ experience of support from colleagues and managers played an important role in facilitating return to work. A respectful attitude from managers created conditions for open dialogue, whereas a lack of managerial support could delay the return to work process. Three-party meetings involving the participant, their manager, and coordinators before return to work were appreciated, providing structured assistance in planning return to work and adjustment needs were taken more seriously. The participants also described support from the coordinator in cases of conflicts or bullying at work.

Yes it (the three-party meeting) was really a good way to change things… I had nagged many times that it does not work as before. "I want a colleague who relieves me" I have said many times before. Now the recruitment process is underway. So, yes, it is sad that sick leave was required. But on the other hand, it’s good to see that it has really influenced the employer as well. This was a signal that could no longer be ignored. (High nonmanual worker, 42 years old woman with exhaustion disorder.)

Interventions from occupational health services (OHS) were criticized by the participants for their time limitations and perceived partiality as OHS were experienced representing the interest of the employer. The participants therefore suggested the inclusion of coordinators within OHS to better support the return to work process for both managers and employees.

### Recognising challenges beyond the person

Participants described that while rehabilitation coordination was important, it was not enough to recover and return to the workplace. Drawing parallels with mental illness and society at large, the participants argued that other initiatives are needed, at a societal level, to promote health. They expressed feelings of guilt about being on sick leave and wished for increased understanding and tolerance for mental illness within society. In this broader societal context, rehabilitation coordination was viewed as just one component, highlighting the need for organizational and societal change to address the challenges associated with mental health and return to work.

#### Stress leads to illness

Participants shared their view that workplaces characterized by streamlined processes and frequent reorganizations contributed to their stress and illness. This was something that they became increasingly aware of and could discuss with the coordinator.

This is our new public disease that must, it must be stopped. I know a company writing that they would have bus drivers who were resistant to stress. Then they were not allowed to put it that way. It should not be allowed to happen. For all people get sick from stress in one way or another. (Intermediate nonmanual worker, 57 years old woman with exhaustion disorder.)

Receiving rehabilitation in PHC was experienced as beneficial and rehabilitation coordination was seen as helpful in improving the conditions for return to work. However, the participants underlined that returning to a stressful work environment undermined a sustainable change; the coordination in itself could not change a stressful workplace. The participants stressed the importance of preventive measures and follow-up by managers, for sustainable return to work. They suggested that proactive efforts and follow-ups by managers could prevent stress and illness, yet it was often neglected.

#### Sick leave may lead to feelings of guilt

The experience of being on sick leave impacted on the participants’ self-esteem and often triggered feelings of guilt. Delays in receiving rehabilitation interventions during sick leave intensified these emotions. Some participants chose to change jobs instead of returning to their existing workplace.

I'm ashamed of my sick leave… I don´t want to meet, I don´t want to see anyone from the organisation I was part of before, because it’s not me. Ím a person who is always two steps ahead and I find solutions, there are no obstacles, and so I end up (on sick leave) and Ím away from work for two years…. If I had come back to the same organisation again, I don´t think I had gained confidence as a good employee. Then it’s just as good to get something else. (Intermediate nonmanual worker, 47 years old woman with exhaustion disorder.)

Feelings of guilt for being on sick leave for mental illness were described by the participants as a considerable obstacle in the rehabilitation coordination process. The lack of knowledge about mental illness in society was also experienced by the participants as leading to a lower understanding and tolerance at work. This was perceived as an additional hindrance for returning to work.

## Discussion

### Main findings

The participants’ experience of rehabilitation coordination was described by the summarising theme *Building a bridge with many bricks between the person and society.* Rehabilitation coordination took place in a complex context, reflected in the theme’s many bricks, creating a bridge between the person and society with the goal of regaining health and the capacity to work. The first three categories described the participants’ perspectives of rehabilitation coordination based on their experience of coordinators, other health care professionals and stakeholders such as employers, the SSIA or OHS. The last category involved rehabilitation coordination as part of a larger societal perspective, experienced as essential for a sustainable working life.

### Comparison with existing literature

Knowledge of rehabilitation coordination was the first cornerstone needed to build the bridge. The present study shows that the participants had difficulties finding information and asking for help because of their symptoms, despite needing rehabilitation coordination, which resulted in delayed support. A qualitative study regarding the ethical aspects of coordination of return to work for people on sick leave with mental health problems presents similar findings: autonomous decisions were hindered by symptoms and there was difficulty understanding the sick leave process [36]. The study also highlighted the risk of unequal inclusion for rehabilitation coordination due to organisational structures and the risk of unequal support due to unclear professional roles. Three-party meetings were considered a way to create consensus among various stakeholders and overcome unclear roles [36]. In the same way, the participants in the present study experienced unclear limits of rehabilitation coordination as several stakeholders were involved. Swedish health care legislation emphasises that those with the greatest need should be prioritised. Furthermore, health care should be based on respect for patients’ self-determination and integrity and should be easily accessible [12]. The present study highlights key areas for improvement in PHC in terms of priorities, self-determination and accessibility for people with mental health problems. Providing accessible information about rehabilitation coordination at PHC centres would facilitate this process.

Some of the bricks in the bridge created by rehabilitation coordination have a person-centred care approach (PCA) as an underlying feature. While PCA is established and promoted in Swedish PHC, it may be difficult to achieve in practice [12,37]. Core values within PCA are to respect and listen to the patient and to show understanding for their self-esteem and will [38]. In Sweden, an initiative to create routines for PCA has been designed from the main component of co-creation of care through partnership between patients, their relatives, and health professionals. By listening carefully to the patient’s narrative and goals, conditions for a co-creation of a health plan are made built on the patient’s strengths and capabilities [39]. In the present study, the participants described a similar collaboration with the coordinators to create sustainable and realistic rehabilitation plans. A clear expression of the participants’ need for PCA was their wish to be seen as persons within their contexts, beyond diagnoses. Most of the participants experienced tactful interplays in line with PCA, however some narratives revealed challenging situations, suggesting a need for calm, clear and tactful communication from the coordinator. An unexpected finding of a non-functioning interplay was described when the participants adapted to the professionals in stressful situations. The present study demonstrated the importance of PCA in creating a functioning rehabilitation coordination as well as the challenges that rehabilitation coordination may face when the interplay did not work. The beneficial experience of person-centred meetings to promote return to work for people with mental health problems has been previously suggested [27,40]. The use of a person-centred dialogue when meeting with the coordinator and the employer provided a common picture of the patient and facilitated return to work [27]. Furthermore, the participants experienced the meeting with the employer as structured and supportive [40]. Using a person-centred dialogue is in line with the findings in the present study showing the importance of a respectful attitude by using PCA.

The bridge built by rehabilitation coordination back to work was experienced as a joint project in the present study. A functioning rehabilitation coordination was dependent on all stakeholders and was always part of a larger context. If the interplay between different stakeholders did not work, obstacles occurred in the sick leave process and return to work could be delayed. Collaboration between different stakeholders with different regulations and interests is challenging and research has shown that collaboration often encounters structural and organisational obstacles [41,42]. The Sherbrooke model [43] has a systems theory perspective which is useful for understanding the parties involved in the sick leave process and return to work, namely the person on sick leave, the workplace, the healthcare system and the social insurance system. Each party acts based on its systems interpretation of reality and collaboration depends on all parties being able to understand the other’s perspective [43]. Recognition of internal organisational factors at PHC centres such as teamwork and leadership has also proved important for the development of rehabilitation coordination [28]. In the present study teamwork was experienced as a powerful way of collaborating where all parties contributed with their knowledge, resulting in better priorities in the sick leave process. External collaboration in identifying support and barriers at work was also described as important by the participants. Collaboration with employers was particularly important, and three-party meetings were experienced as a structured way of planning for return to work. The participants also emphasised that rehabilitation interventions within healthcare were meaningless unless adjustments were made at work. A systematic review of interventions at mental health problems within OHS showed that most studies focused on the individual level and disregarded the work environment or organisational levels [44]. This is remarkable, as the importance of the work environment for employees’ well-being is widely recognised in the literature [45,46]. In the present study the participants confirmed the need for targeted interventions at work and they experienced rehabilitation coordination as a bridge between health care and work to create sustainable conditions for return to work.

Some bricks weakened the bridge built by rehabilitation coordination. One such was the feeling of guilt for being on sick leave. The present study shows that the participants experienced stigma about being on sick leave for mental illness, which could hinder the return to work process. Stigma has been defined by Goffman [47] as negative characteristics that devalue and segregate people from society. People with mental illness often experience public stigma in the form of prejudice and discrimination. The modified labelling theory [48] explains how society’s norms and attitudes are learned during the socialisation process and prejudices and stereotypes are often accepted by those labelled with mental illness diagnoses, leading to internalised stigma. People affected by mental health stigma may have difficulty seeking care and finding work [49]. A qualitative study examining experiences of stigma by people with mental illness shows that psychological interventions facilitated disclosure and coping with stigma [50]. Other studies had similar findings, concluding that disclosing mental health problems reduced internalised stigma and increased quality of life [51,52]. An increased knowledge about mental illness in society might facilitate disclosure from people living with mental health problems, reduce the public stigma surrounding mental illness and lower return to work barriers. The participants’ perspective in the present study highlighted the interconnected nature of individual health challenges with broader societal attitudes and structures. Their call for comprehensive measures at the societal level emphasized the need for a holistic approach that addresses not only individual but also societal factors influencing health and well-being.

### Strengths and limitations

An important strength in the present study is the exploration of experiences of rehabilitation coordination from those having the main role in the sick leave process, namely people on sick leave. The present study adds knowledge about the perspective of people with mental health problems, a subject which is still not sufficiently explored within PHC. A qualitative approach may deepen the understanding of phenomena and the approach facilitated a rich and nuanced description of the participants’ experiences of rehabilitation coordination in the present study. The qualitative approach also highlights the challenges rehabilitation coordination may face. In addition, the authors have extensive experience of qualitative methods, and they used investigator triangulation, meaning that all authors were involved in the analysis process, which strengthened the credibility of the interpretation. Conformability, meaning that interpretations were based on accurate data, was ensured with representative quotations [53].

There are limitations regarding gender balance and recruitment in the present study. Only one participant was male and reaching a better gender balance would have been preferable. Still, more women than men are on sick leave in Sweden [54] and women visit PHC more frequently, largely due to a higher disease burden [55], which is reflected in the study. There is also a risk that people with a negative attitude towards rehabilitation coordination were not informed about the present study, or alternatively declined participation. However, the coordinators were encouraged to inform all eligible participants and among the participants negative experiences of rehabilitation coordination also emerged. Another limitation is the relatively small number of participants in the study, as an appropriate sample size is a way in which to ensure credibility. In qualitative studies, however, it is not the number of participants that is decisive, but the richness of the data and how well the data captures the phenomenon being explored [30,56]. Data were regarded as sufficient after nine interviews had been analysed in an iterative process, at the point when the thematic patterns seemed stable yet with nuanced descriptions. The transferability of the results can also be affected by the number of participants and the context of the study. Even so, there were several similarities with other qualitative studies regarding unclear limits of rehabilitation coordination, the importance of person-centredness, work-oriented interventions and the challenges of stigma that strengthen transferability, all of which are highlighted in the discussion.

## Conclusions

The present study reveals that people with mental health problems experience rehabilitation coordination as a meaningful intervention, building a bridge between them and society, which in turn facilitates return to work. However, to increase accessibility and facilitate autonomous decisions rehabilitation coordination needs to be more visible at PHC centres and information made more easily available.

A person-centred approach is suggested to integrate the needs of the person with resources and requirements at work, increasing the chance of a successful return to work. Future research is needed to further develop meaningful rehabilitation coordination interventions based on the experiences of people on sick leave.
